# Association of oxidative stress, metacognition, and psychopathology in patients with schizophrenia: a case-control study

**DOI:** 10.3389/fpsyt.2026.1899294

**Published:** 2026-07-01

**Authors:** Xiaojuan Hu, Jun Cheng, Shoucui Xia, Lili Ma, Yang Zhang, Li Zhang, Xiaojing Meng, Xulai Zhang, Dongmei Wang, Aiguo Zhang

**Affiliations:** 1 Anhui Medical University Affiliated Psychological Hospital, Hefei, China; 2 Hefei Fourth People’s Hospital, Hefei, China; 3 Institute of Psychology, Chinese Academy of Sciences, Beijing, China; 4 Department of Psychology, University of Chinese Academy of Sciences, Beijing, China

**Keywords:** MAS-A, metacognition, oxidative stress, psychopathology, schizophrenia

## Abstract

**Background:**

Metacognitive deficits are common in schizophrenia (SZ) and may worsen symptoms and impair insight. Oxidative stress (OS) abnormalities have also been reported, but findings are inconsistent, and no study has examined their associations with metacognition and psychopathology.

**Methods:**

This case-control study included 89 SZ patients and 90 healthy controls (HC). OS markers, including superoxide dismutase (SOD), catalase (CAT), malondialdehyde (MDA), and glutathione peroxidase (GPX) were measured. The patient group and healthy control group underwent metacognition was assessed using the abbreviated Metacognitive Assessment Scale (MAS-A) and patients’ symptoms with the Positive and Negative Syndrome Scale (PANSS). Covariates included age, gender, education, BMI, and smoking, illness duration, onset age and medication.

**Results:**

Patients showed significantly lower MAS-A total score and subscale scores (all *p* < 0.01) versus HC. Patients had lower SOD, CAT and GPX (130.69 vs 152.12 ng/L, 2.46 vs 6.62 ng/L, 158.09vs 197.75μmol/L) and higher MDA (9.22vs 7.34μmol/L) than controls (all p < 0.05). Partial correlation revealed that in patients: SOD was negatively correlated with positive/negative/PANSS total and MAS-A decentration scores; CAT was negatively correlated with general pathological/PANSS total scores, and positively correlated with MAS-A total score and its subscales (self-reflectivity, understanding the other’s mind, decentration, mastery), MDA was negatively correlated with negative symptom score and self-reflectivity score, and positively correlated with general pathological score; GPX was positively correlated with most clinical and metacognitive scores. Linear regression revealed SOD, CAT, and GPX significantly associated with the PANSS total score (*β* = -0.119, -6.169, -0.226; all p < 0.05), and with MAS-A total score (*β* = 0.021,2.879 0.049, all p < 0.001).

**Conclusion:**

Schizophrenia patients exhibit OS abnormalities and metacognitive impairments. Greater OS severity correlates with worse metacognition and more severe psychopathology, suggesting OS as a key factor linking these domains.

## Introduction

Schizophrenia (SZ) is a severe mental disorder with a lifetime prevalence of approximately 1% (1). The clinical manifestations mainly include positive symptoms such as hallucinations, delusions, and thinking disorders, negative symptoms such as emotional apathy, social withdrawal, and laziness, as well as cognitive impairment, the latter of which is the main cause of social dysfunction in patients. The cognitive impairment in patients with schizophrenia involves neurocognition, social cognition, and metacognition (2). Its pathogenesis remains unclear, and current theories involve multifactorial contributions from genetics, neurodevelopment, environment, immunity, oxidative stress and neuropsychology.

Oxidative stress(OS), defined as an imbalance between reactive oxygen species production and antioxidant system function, has been repeatedly implicated in schizophrenia. In the nervous system, stressors such as infections or ischemia trigger pro-inflammatory cytokines and oxidative cellular damage (3). A feedback loop exists between neuroinflammation and OS, which amplify each other via pathways like WNT/β-catenin and NF-κB, leading to abnormal levels of metabolites and neurotransmitters associated with SZ symptoms. Studies (4, 5) show that patients with SZ often have significantly reduced peripheral antioxidant levels during the acute phase, and that OS abnormalities may correlate with poor prognosis. Previous studies have identified multiple OS markers associated with clinical symptoms of schizophrenia. SOD is a crucial antioxidant enzyme. In SZ patients, higher SOD levels are associated with poorer information processing speed (6). CAT is an antioxidant enzyme that catalyzes the decomposition of hydrogen peroxide, and it exhibits a significant negative correlation with both the PANSS total score and the negative symptoms scale scores (7). MDA is the final product of lipid peroxidation and reflects the extent of oxidative damage. Higher MDA levels are associated with more severe negative symptoms and slower information processing speeds (8). However, findings remain inconsistent: some studies report decreased SOD level (9), while others find elevated or unchanged levels (10). The discrepancies may be due to medication effects, illness stage, or other confounding factors. OS also contributes to cognitive impairment by damaging neuronal structure and function, disrupting neural plasticity and myelin integrity, and interfering with key signaling pathways (11). Antioxidant therapy has been demonstrated to ameliorate cognitive deficits in SZ model mice (12). Metacognition refers to the ability to monitor and control one’s own cognitive processes, including understanding one’s own and others’ mental states (13–15). Extensive research agrees that patients with SZ exhibit significant metacognitive impairments, considered a core feature of the disorder (16). These deficits manifest as poor awareness, monitoring, and regulation of one’s thinking, as well as difficulties in understanding others’ intentions and mental states (17). Such impairments are closely linked to reduced clinical insight, poorer treatment adherence, worse functional outcomes, and impaired social adaptation (18, 19).

Metacognition is a higher-order cognitive function, yet the neural mechanisms underlying its impairment in SZ remain poorly understood. Current neuroimaging studies point to abnormal functional connectivity in the prefrontal cortex (particularly the dorsolateral and medial prefrontal regions), anterior cingulate cortex, and temporoparietal junctional areas, as well as alterations in brain network connectivity. Importantly, whether metacognitive impairment in SZ is associated with OS has not been investigated.

To address this gap, we conducted a cross-control study to examine the relationship between OS markers, metacognition, and clinical symptoms in patients with SZ.

## Materials and methods

### Participants

A total of 89 patients with SZ and 90 healthy controls were enrolled. Patient participants were diagnosed with SZ by a psychiatrist according to the Diagnostic and Statistical Manual of Mental Disorders Fifth Edition (DSM-5) criteria. All participants were aged between 18–60 years. Patients were hospitalized at Hefei Fourth People’s Hospital between 2023 and 2025. Exclusion criteria included intellectual disabilities, head trauma, severe somatic or neurological diseases, a history of psychoactive substance abuse, comorbid mental disorders, or unstable conditions (e.g., inability to cooperate with the interview). Healthy controls were recruited from the local community and had no personal or family history of psychiatric disorders.

### Clinical evaluation

Clinical evaluations were independently performed by two experienced attending psychiatrists. The severity of psychopathology in patients was assessed using the Positive and Negative Syndrome Scale (PANSS).

### Metacognitive assessment

Metacognitive abilities were evaluated using the abbreviated Metacognitive Assessment Scale (MAS-A), which comprises four subscales: self-reflectivity, understanding the other’s mind, decentration, and mastery. Both the total score and subscale scores were used, with higher scores indicating better metacognitive abilities. A trained psychiatrist conducts face-to-face interviews using the Indiana Psychiatric Illness Interview (IPII), with each session lasting 30–60 minutes and audio recorded. Subsequently, two professionally trained psychiatrists who were unaware of the PANSS results independently scored the interview content using the MAS-A scale based on recordings of the IPII interviews. The intra-rater reliability for the MAS-A was 0.81.

### Measurement of OS markers

Participants were instructed to fast (no food or water) for 6 hours prior to blood sampling. A 5 mL fasting venous blood sample was collected at approximately 7:00 AM. Samples were centrifuged at 3,000 rpm for 5 minutes at room temperature within 1 hour of collection. The supernatant was collected and stored at -80 °C until analysis. Levels of superoxide dismutase (SOD), catalase (CAT), malondialdehyde (MDA), and glutathione peroxidase (GPX) were measured using enzyme-linked immunosorbent assay (ELISA) kits following the manufacturer’s instructions. Equal aliquots of each blood sample were used. All measurements were performed in triplicate by the same researcher, and the average value was used for analysis.

### Statistical analysis

Statistical analyses were performed using SPSS version 22.0. Categorical demographic variables were compared between groups using the chi-square test. Normality of continuous data was assessed using the Shapiro-Wilk test. Normal distributed data were analyzed using independent-samples t-test; non-normal distributed data were analyzed using non-parametric tests as appropriate. Age, gender, education level, disease duration, age of onset, and smoking status were included as covariates in the analysis of covariance (ANCOVA) to compare metacognitive abilities and OS markers between patients and healthy controls.

Person correlation analysis was used for normally distributed data, and Spearman correlation analysis for non-normal distributed data, to examine bivariate associations. Linear regression models were constructed to investigate the associations between OS markers and PANSS total scores as well as total metacognitive total scores, after adjusting for covariates including gender, age, BMI, smoking status, years of education, disease duration, and age of onset. Due to the involvement of multiple comparisons, the study employed the Bonferroni multiple correction to reduce type I errors. Residuals from all four regression models were normally distributed (Shapiro-Wilk test, all *P* > 0.05). Therefore, no logarithmic transformation was required. A two-tailed *P* < 0.05 was considered statistically significant.

## Result

### Demographic and clinical characteristics

A total of 95 patients with SZ were initially enrolled, of whom three were discharged before completing the assessment, two withdrew due to symptom exacerbation, and one refused to cooperate after providing informed consent, resulting 89 patients who completed the study, along with 90 matched healthy controls (HC). As shown in **Table 1**, there were no statistically significant differences between the two groups in age, education level, gender, body mass index, or smoking status (all *P*>0.05).

**Table 1:** Demographic data and general clinical characteristics of the two groups.

Variable	Patients (*n* = 89)		HC (*n* = 90)		*t/χ^2^*	*P*
	Mean	(SD)	Mean	(SD)		
Age (years)	30.69	(9.74)	31.21	(9.75)	0.361	0.719
Education(years)	11.22	(3.01)	11.43	(2.35)	0.517	0.606
BMI (kg/m^2^)	23.61	(4.63)	23.89	(4.06)	0.433	0.666
Gender						
Mail,*n* (%)	42	(47.19)	39	(43.33)	0.269 (*χ^2^*)	0.654
Femail,*n* (%)	47	(52.81)	51	(56.67)
Smoking,*n* (%)	36	(40.45)	33	(36.67)	0.270 (*χ^2^*)	0.603
Age of onset (years)	24.25	(8.23)	–	–	–	–
Disease duration (years)	5.50	(2.33)	–	–	–	–
The PANSS						
Positive	24.36	(9.53)	–	–	–	–
Negative	27.08	(11.02)	–	–	–	–
General psychopathology	35.69	(8.61)	–	–	–	–
PANSS total	87.13	(18.86)	–	–	–	–
Medication (mg/day)	352.25	(52.55)				

HC, healthy control; BMI, body mass index; PANSS, Positive and Negative Syndrome Scale. Medication, Antipsychotic medication (chlorpromazine equivalence).

### Inter-group comparison of metacognition and OS markers

**Table 2** presents the covariance analysis results: compared with the HC group, the patient group had significantly lower scores (self-reflectivity, understanding the others’ mind, decentralization, and mastery) as well as on the MAS-A total (all *P* < 0.001); regarding OS markers, patients showed significantly lower blood levels of SOD, CAT, and GPX (all *P* < 0.05) and significantly higher MDA (*P* = 0.013).

**Table 2:** Comparison of metacognition and OS markers between the two groups.

Variable	Patients (n = 89)		HC (n = 90)		*F*	*P*
	Median	(SD)	Median	(SD)		
MAS-A						
Self-reflectivity	2.98	(1.67)	4.22	(1.59)	25.977	<0.001
Understanding the others’ mind	2.32	(1.65)	3.25	(1.18)	27.025	<0.001
Decentration	0.91	(0.68)	1.56	(0.63)	36.612	<0.001
Mastery	2.69	(1.33)	3.82	(1.13)	370.587	<0.001
MAS-A Total	8.91	(3.05)	12.79	(2.42)	87.427	<0.001
OS index						
SOD (ng/L)	130.69	(41.13)	152.12	(45.79)	10.417	0.001
CAT (ng/L)	2.46	(0.66)	6.62	(4.55)	71.179	<0.001
MDA (μmol/L)	9.22	(5.44)	7.34	(4.32)	6.295	0.013
GPX (μmol/L)	158.09	(39.98)	197.75	(57.72)	28.375	<0.001

HC, healthy control; MAS-A, the abbreviated metacognitive assessment scale; OS, oxidative stress; SOD, superoxide dismutase; CAT, catalase; MDA, malondialdehyde; GPX, glutathione peroxidase.

### Correlation between OS markers in the patients group and clinical symptoms as well as metacognition

**Figure 1:**
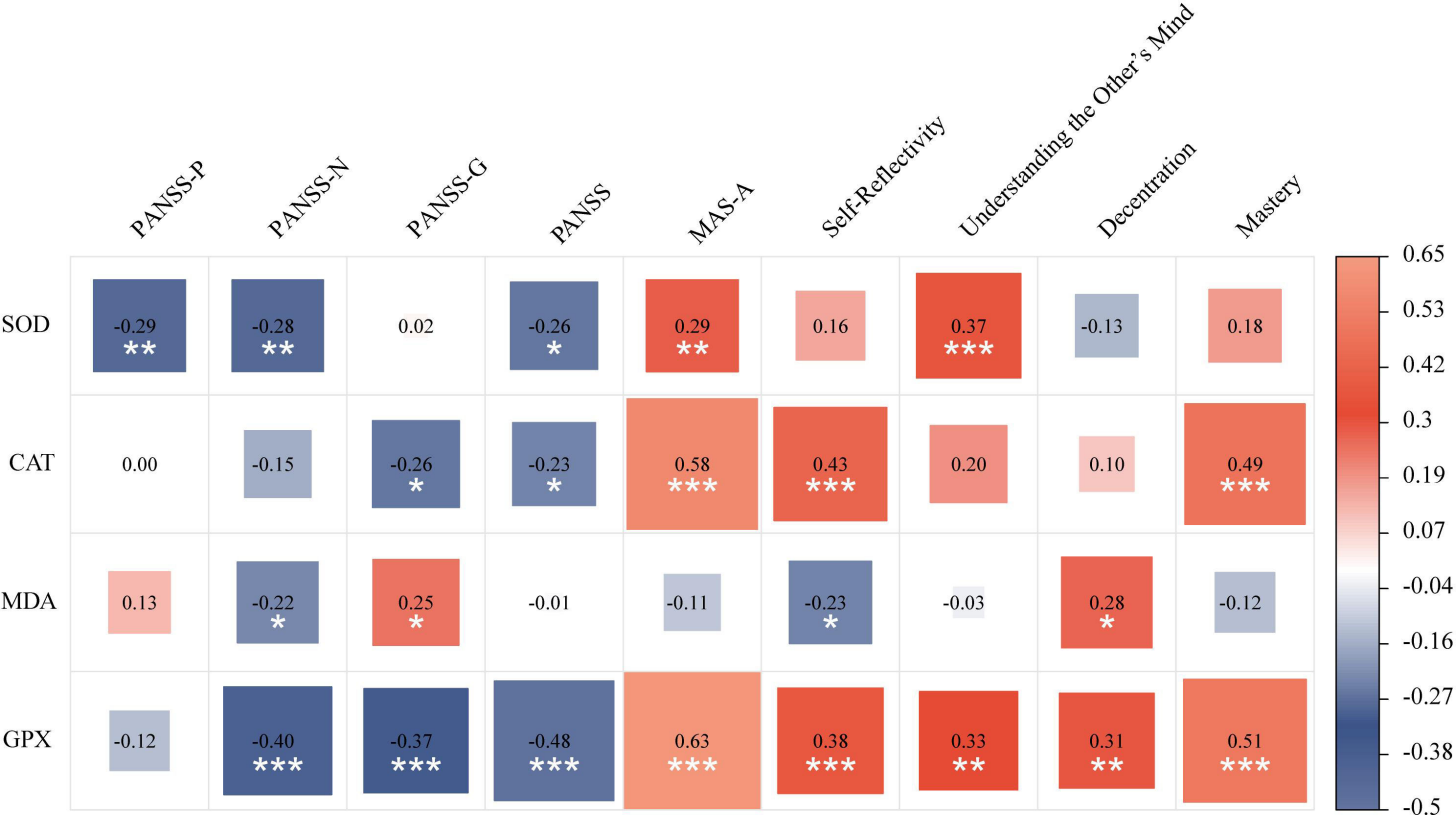
Heatmap showing the correlation between various OS markers and metacognition as well as PANSS scores in the patient group. PANSS, Positive and Negative Syndrome Scale; PANSS-P, Positive syndrome scale; PANSS-N, Negative syndrome scale; PANSS-G, General psychopathology scale. MAS-A, The abbreviated metacognitive assessment scale. *p<0.05, **p<0.01, ***p<0.001.

Partial correlation analysis (controlling for demographic and clinical covariates) revealed that in the patient group, SOD levels were negatively correlated with positive symptom score, negative symptom score, PANSS total score, and positively correlated with MAS-A total score and understanding the others’ mind score. CAT levels were negatively correlated with general pathological score and PANSS total score, and positively correlated with MAS-A total score, self-reflectivity score and mastery score. MDA levels were negatively correlated with negative symptom score and self-reflectivity score, and positively correlated with general psychopathological score and mastery score. GPX levels were negatively correlated with negative symptom score, general psychopathological score, and PANSS total score, and positively correlated with MAS-A total score and all subscales **Figure 1**.

### Multivariate regression analysis of OS markers versus the total PANSS score

**Figure 2:**
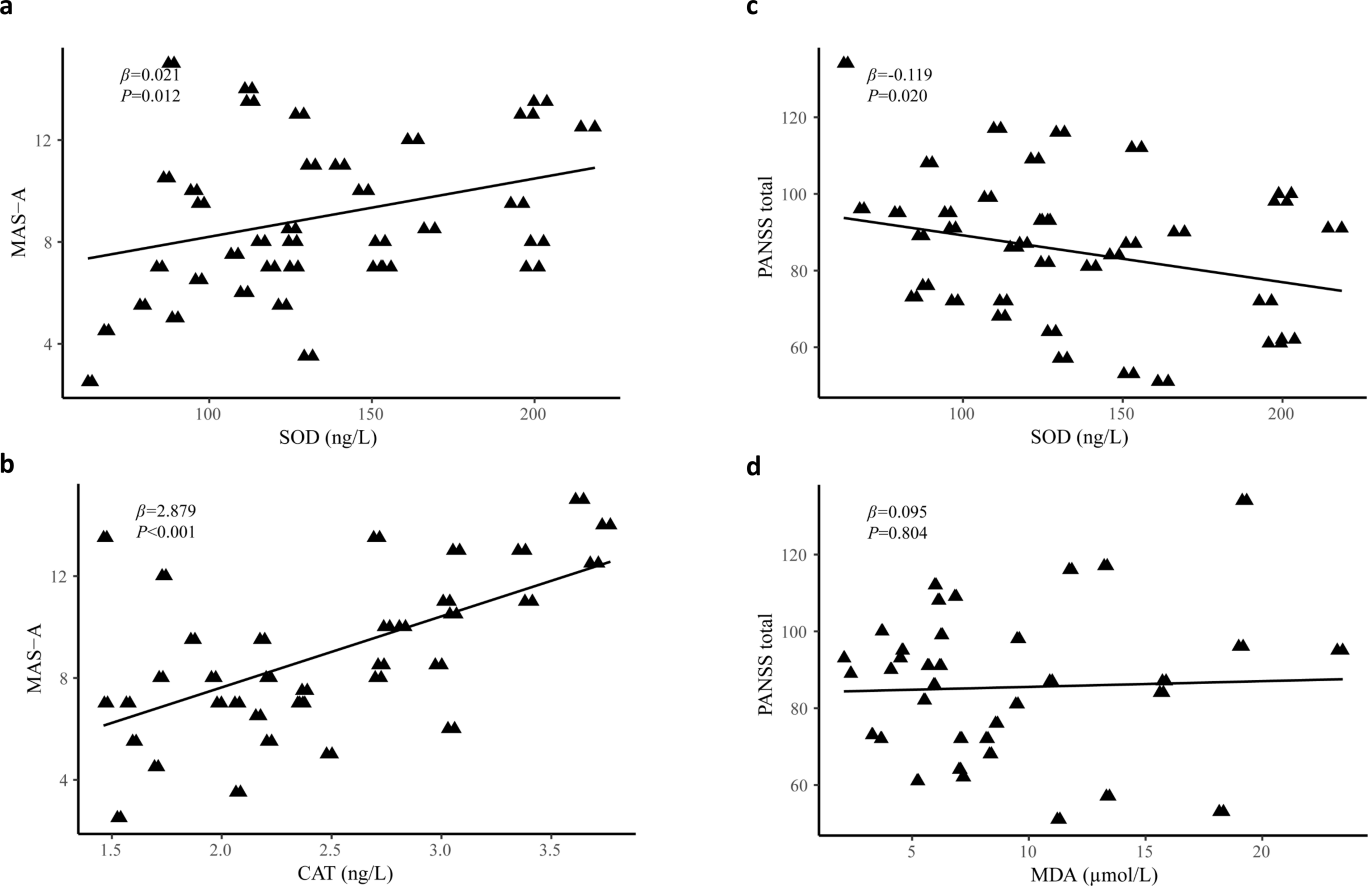
Correlation between OS markers and the total PANSS score. PANSS, Positive and Negative Syndrome Scale; SOD, superoxide dismutase; CAT, catalase; MDA, malondialdehyde; GPX, glutathione peroxidase. **(a)** correlation between SOD and PANSS total score; **(b)** correlation between CAT and PANSS total score; **(c)** correlation between MDA and PANSS total score; **(d)** correlation between GPX and PANSS total score.

Multiple linear regression models, adjusting for age, sex, education, BMI, smoking status, disease duration, and age of onset, further examined these associations. As shown in **Figure 2**, higher SOD, CAT, and GPX levels were significantly associated with lower PANSS total score (SOD: *β* = -0.119, *β*-standardized = -0.264, *P* = 0.020); CAT: *β* = -6.169, *β*-standardized = -0.219, *P* = 0.045; GPX: *β* = -0.226, *β*-standardized = -0.488, *P* < 0.001), whereas MDA showed no significant association with the PANSS total score (*β* = 0.095, *β*-standardized = 0.028, *P* = 0.084).

### Multivariate regression analysis of OS markers versus the MAS-A score

**Figure 3:**
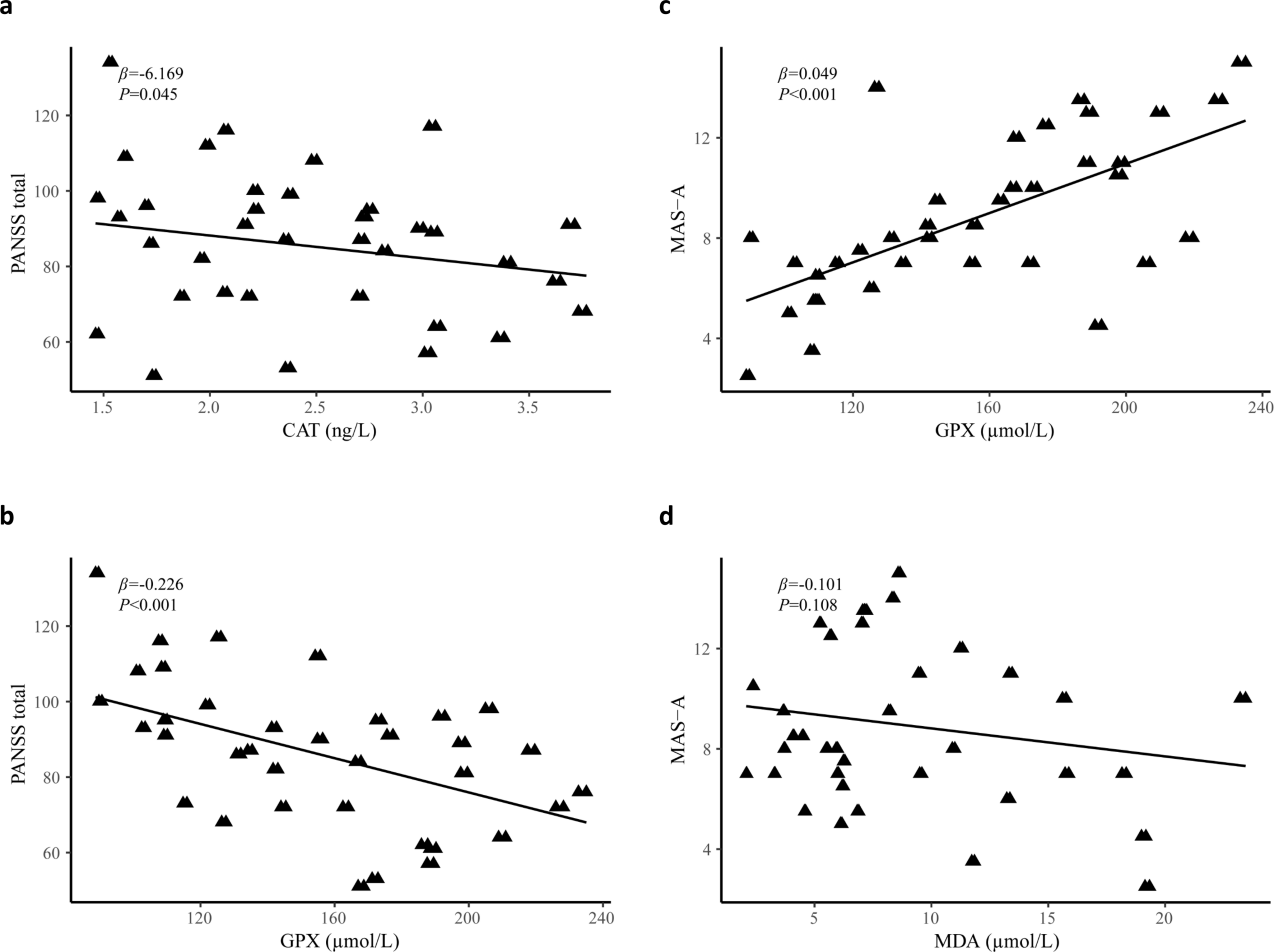
Correlation between OS markers and the total MAS-A score. MAS-A, The abbreviated metacognitive assessment scale.; SOD, superoxide dismutase; CAT, catalase; MDA, malondialdehyde; GPX, glutathione peroxidase. **(a)** correlation between SOD and MAS-A total score; **(b)** correlation between CAT and MAS-A total score; **(c)** correlation between MDA and MAS-A total score; **(d)** correlation between GPX and MAS-A total score.

Regarding metacognitive function (**Figure 3**), higher SOD, CAT, and GPX levels were significantly associated with higher MAS-A total score (SOD: *β* = 0.021, *β*-standardized = 0.286, *P* = 0.012; CAT: *β* = 2.879, *β*-standardized = 0.620, *P* < 0.001; GPX: *β* = 0.049, *β*-standardized = 0.640, *P* < 0.001), while MDA again showed no significant association with MAS-A total score (*P*>0.05).

## Discussion

In this case-control study, after controlling for potential confounders, including sex, age, education level, body mass index, smoking status, disease duration, age of onset, and medication use, patients with SZ showed significantly lower blood levels of SOD, CAT, and GPX and significantly higher MDA level compared with healthy controls. In addition, the metacognitive scale (MAS-A total and all subscales) were significantly lower in the patients group. Importantly, OS markers were significantly correlated with both metacognitive performance and clinical symptoms, suggesting that OS dysregulation is associated with more severe metacognitive deficits and psychopathological alterations. Consistent with numerous previous reports, our study found that SOD, CAT, and GPX levels were significantly reduced in SZ patients relative to healthy individuals, further supporting the hypothesis of impaired antioxidant function in SZ. However, some studies have reported no difference or even decreased SOD levels (10). These discrepancies may attributed to differences in sample characteristics (e.g., first-episode vs. chronic), illness duration, and antipsychotic treatment, as well as methodological variations in sample size and inclusion criteria. Nevertheless, as the body’s primary antioxidant defense, SOD appears to be markedly comprised in SZ. In contrast, changes in CAT, MDA, and GPX have been more consistently observed across studies, with reduced CAT and GPX and elevated MDA. As the principal antioxidant enzymes in the body’s antioxidant system, SOD converts O_2_^-^ to H_2_O_2_, while GPX and CAT further convert H_2_O_2_ to H_2_O and O_2_ (20). In the early stages of SZ, excessive H_2_O_2_ and other peroxides are produced. When the antioxidant capacity is insufficient, free radicals induce lipid peroxidation, causing cell and tissue damage, which in turn impairs neuronal plasticity, neural signal transmission, and n membrane protein-dependent neurotransmitter uptake – mechanisms that implicate free radicals in the pathogenesis of the disorder (21–23). To counteract free radicals damage, the body may consume more SOD and GPX, leading to reduced measured activities. Additionally, under OS, changes in the kinetic parameters of GPX change may prevent its active center (selenocysteine) from effectively binding with glutathione, further diminishing its activity (24). Our second finding is that OS markers were significantly correlated with PANSS scores, which is highly consistent with most previous studies (8, 25–27). Specifically, SOD levels showed a stronger association with positive symptoms, while GPX levels correlated with negative symptoms, general pathological symptoms, and the total score. SOD is an early marker of OS dysregulation, with abnormalities typically emerging in the early disease stage, while GPX abnormalities occur later. Early-stage SZ is often characterized predominantly by positive symptoms; as the disease progresses, negative and cognitive symptoms become more prominent (most of which are captured by the general pathological subscale). This pattern suggests that OS levels may serve as predictive indicators of disease stage and clinical progression. Although OS may not be the primary etiology of SZ, elevated OS likely contribute to disease exacerbation and poor prognosis (28, 29).

To our knowledge, this is the first study to report a significant association between OS abnormalities and more severe metacognitive impairment in SZ. Drawing on prior neurocognitive research, we propose several potential mechanisms. First, OS dysregulation has been strongly linked to neurocognitive deficits in SZ patients. The production of excessive reactive oxygen species (ROS) and subsequent cellular damage plays a critical role in neuronal development. Increased ROS levels can induce damage via lipid peroxidation, DNA damage, and regulation of cell-related genes, leading to impairment of specific neuronal circuits and resulting in neurocognitive deficits, particularly in working memory and executive functions (6, 30, 31). The study found that 76.1% of general cognitive decline (G-CoDe) observed in patients with methamphetamine-induced psychosis (MAP) could be attributed to a combination of enhanced oxidative toxicity, diminished antioxidant defense capacity, frequency of psychotic episodes, and methamphetamine (MA) dosage. In MAP patients, myeloperoxidase (MPO) levels were significantly correlated with G-CoDe. MA administration induces mild cognitive impairment by activating OS pathways while inhibiting antioxidant pathways (32). The pathogenesis of MAP closely resembles that of SZ, making it a well-established animal model for SZ research (33). Therefore, abnormalities in the OS pathway are closely associated with cognitive impairments in SZ. Second, neurocognition provides the foundational system for metacognitive monitoring, as metacognition is a higher-order cognitive ability closely intertwined with neurocognitive functions. Working memory serves as an essential “computational resource” for metacognitive processing: individuals must retain the content of their current thought working memory to monitor and evaluate them. Palmer et al. suggested that superior working memory may act as a compensatory mechanism, enabling patients to perform metacognitive monitoring of their psychotic thoughts (34). Executive functions provide the fundamental capacity for evaluating and regulating one’s own thinking. Thus, OS imbalance in SZ patients may lead to neuronal circuit damage, resulting in neurocognitive dysfunction and, consequently, impaired metacognitive abilities due to compromised resources and foundations for support.

## Conclusion

This study demonstrated that patients with SZ have significantly lower levels of blood antioxidant enzymes (SOD, CAT, GPX), higher levels of the lipid peroxidation product MDA, and marked impairments in metacognitive function compared to healthy controls. Importantly, this is the first report of a significant correlation between OS markers and metacognitive deficits in SZ: greater disruption of the OS system is associated with more severe metacognitive impairment and psychopathology. The differential correlation patterns between PANSS scores—SOD more strongly associated with positive symptoms, and GPX more closely correlated with negative symptoms and general psychopathology— suggest that OS profiles may reflect disease stages and clinical progression, supporting their potential as predictive biomarkers (28, 29). As neurocognition provides the supportive substrate for metacognitive monitoring, its decline reduces the computational resources necessary for metacognitive processing, ultimately resulting in comprehensive metacognitive impairment. In patients with SZ, abnormal oxidative stress may impair metacognitive functions by damaging fundamental cognitive abilities such as working memory and executive functions (35). However, this is a cross-sectional study and cannot establish causal relationships; we will conduct further research to investigate the causal relationships and mediating effects among these three variables. This study provides novel empirical evidence linking molecular abnormalities to higher-order cognitive functions in patients with SZ and offers a theoretical rationale for future intervention strategies targeting OS to improve metacognitive outcomes. This study has several limitations. First, it is a cross-sectional study and thus cannot establish causal relationships. Second, we did not differentiate between patients with first-onset SZ and those with relapse. Third, we did not perform stratified analyses by drug type. In future studies, we will optimize the research design by incorporating neurocognitive data to further clarify the associations between OS, neurocognition, and metacognition.

## Data Availability

The original contributions presented in the study are included in the article/supplementary material. Further inquiries can be directed to the corresponding author.
